# Walkability and self-rated health in primary care patients

**DOI:** 10.1186/1471-2296-5-29

**Published:** 2004-12-02

**Authors:** James Rohrer, JR Pierce, Anne Denison

**Affiliations:** 1 Department of Family and Community Medicine, Texas Tech University Health Sciences Center, Health Services Research, 1400 Wallace Blvd. Amarillo, Texas, 79106, USA; 2 Womens' Health and Research Institute, 1400 Wallace Blvd, Amarillo Texas 79106, (PH) 806-354-5786, (FX) 806-356-5908; 3 Department of Internal Medicine, Texas Tech University Health Sciences Center, Amarillo, Texas USA; 4 Texas Tech University Health Sciences Center, Amarillo, Texas USA

## Abstract

**Background:**

The objective of this study was to investigate the relationship between perceived walkability and overall self-rated health among patients who use community-based clinics.

**Methods:**

A cross-sectional survey was distributed to a convenience sample in three community clinics. Forms were completed by 793 clinic patients. Multiple logistic regression analysis was to control for the effects of demographic variables and lifestyles.

**Results:**

Perceiving the availability of places to walk was related to better self-rated health. The most important places were work (OR = 3.2), community center (OR = 3.12), park (OR = 2.45) and day care (OR = 2.05). Respondents who said they had zero (OR = .27) or one (OR = .49) place to walk were significantly less healthy than persons who said they had five or more places to walk.

**Conclusion:**

Persons who perceived that they had no place to walk were significantly less healthy than persons who thought they had at least one place to walk (OR = .39). Support for walkable neighborhoods and education of patients about options for walking may be in the best interests of community medicine patients.

## Background

Much recent interest and research has been directed at the relationship between one's health and where one lives [1]. A number of measures of health (including all-cause mortality [2,3], low infant birth weight [4], unintentional childhood injuries [5], hospitalization for asthma [6], and the risk for certain arboviral diseases [7,8] have been associated with neighborhood effects that are independent of demographic health markers such as age, gender, race, and poverty. The neighborhood environment appears to exert health effects independent of or in addition to the health behaviors of the neighborhoods inhabitants [9].

Neighborhoods where walking is convenient might encourage their inhabitants to exercise. Indeed, having convenient places to walk in the neighborhood has been related to the proportion of persons in the neighborhood who met current activity recommendations [10] and with a decreased prevalence of overweight [11]. Neighborhoods with convenient places to walk are characterized by good "walkability." Walking is now recommended for the prevention and treatment of many common diseases, such as hypertension, diabetes, coronary heart disease, osteoporosis, colon cancer, and obesity [12]. Therefore, one would postulate that neighborhoods characterized by good walkability would be inhabited by healthier residents.

The hypothesis that perceived walkability is directly related to an individual's overall self-rated health has not previously been investigated. The purpose of this project was to test that hypothesis in patients attending community-based clinics. Self-rated health has been shown to accurately predict overall health as measured by other more traditional measures of health [13].

## Methods

A cross-sectional survey was used to test the hypothesis that patients with good self-rated health perceived the neighborhoods in which they live as having good walkability, and that this effect was independent of demographic characteristics and lifestyle habits that would predict good health. The sample was drawn from three clinics that primarily serve low-income populations. Clients, parents (if the patient was a child) and accompanying visitors were asked to complete surveys and drop them into a box. Participation was voluntary. A total of 1471 surveys were distributed with an overall return of 825 (56.1%) surveys of which 793 (54%) met eligibility parameters. Pregnant women and persons under age 18 were excluded. Assuming 80% power, p < .05, 20% poor health among the persons inhabiting neighborhoods with good perceived walkability and 30% poor health among persons inhabiting neighborhoods with poor perceived walkability, 626 cases were needed to test the hypothesis. Completed forms were received from a total of 793 persons.

Return rates varied by clinic. Clinic 1 is a university-based family medicine clinic providing a full range of primary care services to cross-generational clients. It is staffed by family medicine physicians and residents. Census was approximately 85 clients daily, of which, less than 5 % were non-English speaking. Clinic personnel distributed 500 survey forms over an eight week period with an 80.8% return rate.

Clinic 2 serves women and children, providing obstetrical, well care (including immunizations), and acute care services to a targeted high-risk, low socioeconomic sub-population. It is staffed by pediatric and OB-GYN physicians and residents. Approximately 30% of the clinic clients do not speak English. A total of 471 surveys were distributed over a period of 18 weeks with a return of 37.4%. Both the large number of obstetrical patients (ineligible for survey) and percentage of non-English speaking clients contributed to the low return rate.

Clinic 3 provides primary care services to a population of indigent adults meeting residential and income screening requirements. It is staffed by internal medicine physicians and residents. A total of 500 surveys were distributed in this clinic over a ten week period with a return of 42.6%.

The dependant variable for the study was self-rated health. Subjects were asked whether in general they would say their health was excellent, very good, good, fair or poor. Excellent, very good, and good responses were combined to form a category called 'good health' while fair and poor comprised 'poor health'.

Leyden's scale of walkability was modified for a US population for this study[11] by dropping the terms newsagent, chemist, and crèche. The question reads: "A lot of people are very dependent on a car these days to get where they want to go. If you or another family member wanted to which of the following could you walk to without too much trouble. Circle all you could walk to without too much trouble." Possible answers were a local corner shop, a church, a park, a local school, a community center or recreation center, a day care center, a drug store, a bar or pub, the place that I work, or "none of the above. It is really hard to go anywhere without a car."

Both demographic characteristics and lifestyle variables were used to adjust the associations between perceived walkability and self-rated health. Lifestyle variables were: numbers of fruits and vegetables eaten per day (zero, one, two, three, four, five or more), smoking status (not a smoker, smokes one-20 cigarettes per day, smokes more than 20 per day), days of physical activity per week that involve at least 20 minutes of exercise (zero, one, two, three, four, five or more), and obese (yes vs no). Obese was defined as body mass index (BMI) >30. BMI was computed from self-reported height and weight.

Demographic variables were race/ethnicity (Hispanic, non-Hispanic white, non-Hispanic black, non-Hispanic Asian, non-Hispanic other), gender, age category, marital status (married vs. not), and highest level of education achieved.

Chi square tests were performed to test for any unadjusted associations between self-rated health and each categorical independent variable. Multivariate logistic regression modeling was employed to determine if associations between perceived walkability and self-rated health remained significant after adjustment for demographic and lifestyle variables. Separate logistic regression models were run for each variable. Statistical analysis was performed using EpiInfo 3.2.2.

## Results

The question about self-rated overall health was answered by 793 respondents. Of these, 67 percent were classified as healthy because they said their health was excellent, very good, or good. The remainder was classified as having poor health because they said their health was fair or poor.

The typical respondent was non-Hispanic white, female, had at least a high school education. The sample was evenly spread across age groups. About half were married and about half were not married (Table 1).

**Table 1:** Association between good self-rated health and demographic variables (chi-square)

Variable	Overall Percent	Percent Healthy	Pct Not Healthy	P
Age				0.0717
18–25	17.0	77.6	22.4	
26–35	21.6	66.5	33.5	
36–45	17.0	64.2	35.8	
46–55	19.1	64.0	36.0	
56–65	12.3	69.1	30.9	
Over 65	12.8	60.4	39.6	
Race/ethnicity				0.0303
Hispanic	15.5	58.5	41.5	
NH* asian	.5	75.0	25.0	
NH* black	7.8	54.8	45.2	
NH* white	74.9	69.9	30.1	
NH* other	1.3	70.0	30.0	
Gender				0.4852
Male	18.7	64.2	35.8	
Female	81.3	67.6	32.4	
Education				0.0011
Less than high school	9.0	54.9	45.2	
High school	37.8	62.0	38.0	
Some College	37.7	69.6	30.4	
College degree	15.5	79.7	20.3	
Married				<0.0001
Yes	49.8	74.7	25.3	
No	50.2	59.3	40.7	

*NH = Non-Hispanic

Unadjusted relationships between demographic variables and self-rated health are shown in Table 1. Statistical significance was determined using chi-square tests. As expected, the percent healthy declined with age. Non-Hispanic white respondents were more likely to report good self-rated health than Hispanics or non-Hispanic blacks. The percent healthy increased with level of education and married persons were more likely to be healthy. No significant difference was seen between male and female subjects in self-rated health.

The most common number of days of exercise per week was zero (34.9 percent). Most people said they ate at least one fruit or vegetable each day, though only less than half had three or more. The percent obese was 43.6. Over 70 percent were non-smokers (see Table 2).

**Table 2:** Association between good self-rated health and lifestylevariables (chi-square)

Variable	Overall Percent	Percent Healthy	Pct Not Healthy	p
Days of exercise per week				0.0104
Zero	34.9	62.9	37.1	
One	8.0	52.4	47.6	
Two	16.6	67.9	32.1	
Three	18.8	72.3	27.7	
Four	5.8	80.4	19.6	
Five or more	16.0	71.4	28.6	
Fruit and vegetables				0.1522
Zero	15.2	62.5	37.5	
One	19.6	70.8	29.2	
Two	27.6	68.7	31.3	
Three	20.1	66.5	33.5	
Four	9.9	56.4	43.6	
Five or more	7.6	75.0	25.0	
Obese				<0.0001
Yes	43.6	58.1	41.9	
No	56.4	73.8	26.2	
Smoking Status				0.1496
Non-smoker	72.0	68.9	31.1	
One – 20 per day	26.1	61.8	38.2	
Over 20 per day	1.9	60.0	40.0	

Self-rated health was related to lifestyle variables (Table 2). Persons who exercised more times per week were more likely to report good self-rated health. Obese persons were less likely to be healthy. Smoking and consumption of fruit and vegetables were not significantly related to self-rated health, though more smokers reported poor health.

The association between perceiving particular places to walk and self-rated overall health is shown in Table 3. Respondents typically said they could not walk to the places included in the walkability scale. The highest percents were corner store (43.1), park (44.0), and school (40.9). When the association between particular places to walk and self-rated health was adjusted for the demographic and lifestyle variables, eight were significant: workplace (OR = 3.2, p = .0011), church (OR = 1.76, p = .0031), community center (OR = 3.12, P = .0019), corner store (OR = 1.71, p = .0032), day care (OR = 2.05, p = .0216), drug store (OR = 1.88, p = .0055), park (OR = 2.45, p < 0.0001), and school (OR = 1.86, p = .0008).

**Table 3:** Association between Good Self-Rated Health and Places to Walk (N = 793)

Variable	Overall Percent	Percent Healthy	Pct Not Healthy	p	Ajusted Odds Ratio (Confidence Interval)*	p
		67.0	33.0			
Pub				0.5043		
Yes	10.1	72.5	27.5		1.38 (.77–2.48)	0.2750
No	83.9	66.2	33.8		reference	
Workplace				0.0146		
Yes	8.7	82.6	17.4		3.20 (1.5955–6.4271)	0.0011
No	85.1	65.3	34.7		reference	
Church				0.0010		
Yes	32.2	75.7	24.3		1.76 (1.21–2.57)	0.0031
No	61.8	62.2	37.8		reference	
Community Center				0.0030		
Yes	9.6	84.2	15.8		3.12 (1.52–6.38)	0.0019
No	84.4	64.9	35.1		reference	
Corner Shop				0.0002		
Yes	43.1	74.6	25.4		1.71 (1.20–2.44)	0.0032
No	50.8	60.3	39.7		reference	
Day Care				0.0176		
Yes	11.3	80.0	20.0		2.05 (1.11–3.78)	0.0216
No	82.6	65.0	35.0		reference	
Drug Store				0.0037		
Yes	21.7	77.3	22.7		1.88 (1.20–2.93)	0.0055
No	72.3	63.7	36.3		reference	
Park				<0.0001		
Yes	44.0	76.8	23.2		2.45 (1.69–3.55)	<0.0001
No	49.9	58.1	41.9		reference	
School				<0.0001		
Yes	40.9	76.2	23.8		1.86 (1.29–2.67)	0.0008
No	53.1	59.6	40.4		reference	

*adjusted for marital status, age, gender, obesity, smoking status, days of exercise per week, educational level, race/ethnicity using multiple logistic regression (N = 775)

Table 4 shows the association between the number of places to walk and self-rated health. Nearly 30 percent of respondents reported that they had no places to which they might walk. Using five or more places to walk as the reference category and adjusting for demographic variables and lifestyle, persons with no places to walk had lower odds of being in good health (OR = .27, p < 0.0001). Persons who had only one place to walk also were not as healthy (OR = .49, p = .0257). The other levels of walkability were not significantly different from five or more places.

**Table 4:** Association between Good Self-Rated Health and Number of Places to Walk

Variable	Overall Percent	Percent Healthy	Pct Not Healthy	p	Ajusted Odds Ratio (Confidence Interval)*	p
		67.0	33.0			
Walk category				<0.0001		
None	27.9	51.1	48.9		.27 (.16–.47)	<0.0001
One	14.6	65.5	34.5		.49 (.26–.92)	0.0257
Two	11.6	75.0	25.0		.76 (.37–1.54)	0.4414
Three	12.5	72.7	27.3		.66 (.34–1.30)	0.2281
Four	10.2	72.8	27.2		.72 (.35–1.45)	0.3505
Five +	17.2	80.1	19.9		reference	
Missing	6.1	68.8	31.3		.59 (.26–1.30)	0.1898
No place to walk				<0.0001		
Yes	27.9	51.1	48.9		.39 (.27–.57)	<0.0001
No	72.1	73.1	26.9		reference	

*adjusted for marital status, age, gender, obesity, smoking status, days of exercise per week, educational level, race/ethnicity using multiple logistic regression (N = 775)

Also shown in Table 4 is the result of a multiple logistic regression analysis in which perceived walkability is scored simply as none versus more than none. In this model, the adjusted odds ratio for perceived walkability was .39 (p < 0.0001)

## Discussion

Studies of physical activity in public health may be classified according to the intensity of physical activity on which they focus (the "meet recommended guidelines" group versus the "any activity will do" group). Meeting recommended physical activity guidelines is related to better self-rated health [14]. Certainly, more fitness is better, but arguments can be made against setting high standards for physical activity in the population. Foremost among these is the observation that effective interventions to promote high levels of fitness in the general population may not be available. Harlan reported that a brief intervention in primary care was not effective [15], as did Yeazel [16]. In contrast, Long's study supported primary care based promotion of physical activity [17] and Eakin also offered support [18]. Overall, most studies of the elderly do not show that exercise reduces disability [19].

If promotion of intensive physical activity is problematic, then more moderate activity might be a more reasonable goal. Simple walking, for example, reduces the cost of medical care for the elderly [20] and interventions have been developed to increase walking [21]. Besides, sedentary adults may not be able to accurately recall the intensity of physical activity, casting doubt on the accuracy with which it can be measured [22].

In addition to valuing leisure-time walking, researchers and clinicians should take into account the benefits of work related activity and housework [23]. So called "lifestyle" interventions are more cost-effective than supervised center-based exercise [24]. In short, encouraging moderate physical activity is important. Successfully doing so in the most sedentary and unfit portion of the population (the bottom 20 or 25 percent) would generate large gains in population health [25].

One approach to encouraging moderate physical activity is to increase walkability. The convenience of places to exercise is widely recognized to be important; adults generally support local policies that increase the availability of places to exercise [26-28]. Walkability in Georgia was investigated by Powell et al, using the state-wide Behavioral Risk Factor Surveillance System (BRFSS) [10]. Over 90 percent of Georgians reported that they knew of a place where they felt safe walking. The most common place was the respondent's neighborhood (32%). About half said that they could get to their walking place in less than ten minutes. A direct relationship was found between convenience of the walking place and the proportion of respondents meeting current activity recommendations. Better health should result from more walking, but this relationship was not tested in the Georgia study.

Our purpose was to investigate the effect of perceived walkability on the health of patients attending community based clinics. The results should not be interpreted as being relevant to all persons in the community or even to all persons who live in neighborhoods they perceive to be unwalkable, but only to patients attending clinics that serve a disproportionate share of disadvantaged persons. This is important to public health because the success of strategies designed to improve in overall community health will have to focus on the disadvantaged people who bear the greatest burden of poor health. We believe that more studies of disadvantaged clinic populations such as this one are needed in the public health literature.

## Conclusions

The study reported here of low income primary care patients confirms the relationship between perceptions of convenient walking locations and self-rated health. Health status is measured by just one question, a practice which has become increasingly common in the public health literature[29,30]. However, since the study employs a cross-sectional design the relationships between perceived walkability and good health might be due to an omitted third variable, such as a tendency to look for excuses for inactivity among persons of poor health. Or a tendency for negative persons to report no places to walk may have influenced the results of this study. In addition, since a large proportion of our respondents were female, the results might be less generalizable to male populations.

Another limitation of the study is the subjective nature of the walkability measure. Perceptions of walkability may not be accurate and thus objective assessment of walkability might have led to a different conclusion. However, since our purpose was to investigate the relationship between perceptions of walkability and self-rated health, the results are valid for this sample. If community medicine patients are incorrect in their perceptions of neighborhood walkability, then public health education campaigns and personal health education in clinics can inform them about options for walking in their neighborhoods and how they might overcome any perceived barriers.

Our results also may have been influenced by the context from which subjects were drawn. Amarillo is located in the Panhandle of Texas. Most of the year the climate is mild, but during the summer season temperatures can average over 90 degrees. In some neighborhoods, crime rates are above the national average and walking after dark might be worrisome. Furthermore, cities in West Texas are designed for automobile traffic, to the detriment of pedestrians and bicyclists. Furthermore, the vast open spaces in the region make it impossible to reach many locations by foot. Accordingly the, culture of the area has not evolved to be supportive of walking. Therefore, our results may not be generalizable to communities where walking has historical been a visible aspect of the culture.

Despite these limitations, the results of this analysis are intriguing and warrant further investigation using a prospective study design. The evidence presented suggests that support for walkable neighborhoods and health education about options for walking in their neighborhoods may be in the best interests of community medicine patients.

## Competing interests

The author(s) declare that they have no competing interests.

## Authors' contributions

JER planned the project, analyzed the data and wrote the first draft of the manuscript. JRP contributed comments and revisions to the manuscript. AD organized data collection and wrote a portion of the manuscript.

## Pre-publication history

The pre-publication history for this paper can be accessed here:


